# A Self-Sustained Wireless Multi-Sensor Platform Integrated with Printable Organic Sensors for Indoor Environmental Monitoring

**DOI:** 10.3390/s17040715

**Published:** 2017-03-29

**Authors:** Chun-Chang Wu, Wen-Yu Chuang, Ching-Da Wu, Yu-Cheng Su, Yung-Yang Huang, Yang-Jing Huang, Sheng-Yu Peng, Shih-An Yu, Chih-Ting Lin, Shey-Shi Lu

**Affiliations:** 1 Graduate Institute of Electronics Engineering, National Taiwan University, Taipei 10617, Taiwan; junwu0208@hotmail.com (C.-C.W.); d99943047@ntu.edu.tw (W.-Y.C.); davidwuct@gmail.com (C.-D.W.); sycjohn19910205@gmail.com (Y.-C.S.); Zizou.ee99@g2.nctu.edu.tw (Y.-Y.H.); sy2213@gmail.com (S.-A.Y.); timlin@ntu.edu.tw (C.-T.L.); 2 Department of Electrical Engineering, National Taiwan University of Science and Technology, Taipei 10617, Taiwan; tim82518@gmail.com (Y.-J.H.); sypeng@mail.ntust.edu.tw (S.-Y.P.)

**Keywords:** self-sustained, printable organic sensors, sensing platform, SoC, power-gating

## Abstract

A self-sustained multi-sensor platform for indoor environmental monitoring is proposed in this paper. To reduce the cost and power consumption of the sensing platform, in the developed platform, organic materials of PEDOT:PSS and PEDOT:PSS/EB-PANI are used as the sensing films for humidity and CO_2_ detection, respectively. Different from traditional gas sensors, these organic sensing films can operate at room temperature without heating processes or infrared transceivers so that the power consumption of the developed humidity and the CO_2_ sensors can be as low as 10 μW and 5 μW, respectively. To cooperate with these low-power sensors, a Complementary Metal-Oxide-Semiconductor (CMOS) system-on-chip (SoC) is designed to amplify and to read out multiple sensor signals with low power consumption. The developed SoC includes an analog-front-end interface circuit (AFE), an analog-to-digital convertor (ADC), a digital controller and a power management unit (PMU). Scheduled by the digital controller, the sensing circuits are power gated with a small duty-cycle to reduce the average power consumption to 3.2 μW. The designed PMU converts the power scavenged from a dye sensitized solar cell (DSSC) module into required supply voltages for SoC circuits operation under typical indoor illuminance conditions. To our knowledge, this is the first multiple environmental parameters (Temperature/CO_2_/Humidity) sensing platform that demonstrates a true self-powering functionality for long-term operations.

## 1. Introduction

Wireless sensor network (WSN) and system-on-chip (SoC) technologies have been widely used in many Internet-of-Things (IoT) applications [1,2] that have significantly improved our daily lives. Among the different IoT applications, environmental monitoring is of importance because of living comforts. To create a comfortable indoor environment for human activities, it is crucial to monitor surrounding air quality parameters, such as temperature, humidity and concentration of carbon dioxide (CO_2_) [3,4,5]. Without the information of these parameters, people may be at high risk of respiratory infection or health disorders. Consequently, the integration of temperature, humidity, and CO_2_ sensors into one environmental monitoring platform is of much interest to provide better life quality.

Optical sensors [6], piezoelectric devices [7], carbon nanotubes [8], metal oxide devices [9] and polymer composites [10] are several types of commonly used gas sensors for humidity and CO_2_ detection. Most of these commercial sensors operate at high temperature or employ infrared transceivers. The required interface circuits are thus complicated with tremendous power consumption [11,12,13,14,15,16,17]. Therefore, such sensing devices are expensive and typically powered from power cords or batteries. However, power line deployments as well as battery replacements are inconvenient and costly. To circumvent these drawbacks, a low-power and low-cost environmental monitoring system for air quality control is developed and presented in this paper.

**Figure 1:**
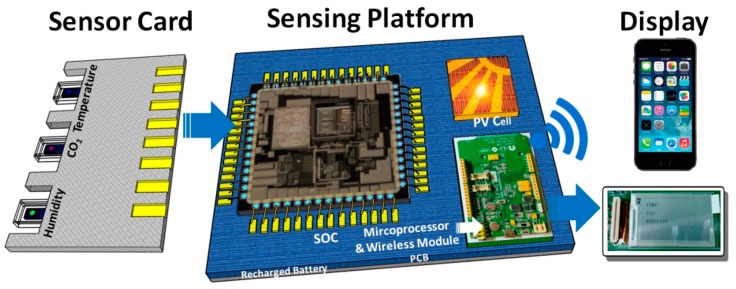
The application scenario of the developed sensing platform with a sensor card that includes sensors for temperature, CO_2_, and humidity detection.

In an environment monitoring system, power consumption is one of the most critical and challenging design considerations in developing sensors as well as relevant interface/control circuits. To develop a low-power and low-cost environmental monitoring system, polymer composites are adopted for humidity and CO_2_ sensing because of their high sensitivity, reasonable response time, low manufacturing cost, and low power consumption for operations [18]. Additionally, functional groups on polymer backbones can be adjusted by co-polymerization or structure derivations for different sensing targets [19]. On the other hand, a commercial thermistor is exploited in the developed sensing platform for temperature sensing. All these sensors, including a commercial thermistor (The waveform of temperature is followed by a linear equation from 20 °C to 70 °C. The measurement results are shown in Figure S1) and two types of polymer sensors for humidity and CO_2_, are integrated on a sensor card so that they can be conveniently replaced or substituted by other state-of-the-art sensors developed in the future. Furthermore, to incorporate multiple sensors in a power-efficient and economical environmental monitoring platform, the system-on-chip (SoC) approach [20,21,22,23,24,25,26] is adopted to integrate the power management unit (PMU), analog sensing front-end (AFE), analog-to-digital convertor (ADC), and digital controller on a single chip. A control scheme employing the power gating method with short duty cycles is utilized to achieve ultra-low average power consumption. The developed monitoring SoC system is powered by a rechargeable Li-ion battery that can be charged by a photovoltaic transducer through an on-chip integrated charger circuit. The sensed environmental data can be displayed on a commercial electronic-paper or via a web browser by transferring to a commercial wireless module and a cloud server. In the SoC system, the charger harvests energy from commercial dye sensitized solar cells (DSSC) under typical indoor luminance conditions. The capabilities of sensor replacement, self-powering, power-gating with proper timing control, and wireless communication enable this system to be self-sustained and low-cost. This developed platform can be applied to different sensing scenarios. Figure 1 shows one of the application scenarios of the developed sensing platform with a sensor card.

## 2. Materials and Methods

### 2.1. Printable Organic Sensor Design and Fabrication

As mentioned previously, to realize the efficient self-powered environmental monitoring system, primary considerations for sensors are fabrication cost and operating power consumption. Since organic sensing materials can be printed by inexpensive ink-jet printing processes at room temperatures, they are adopted to implement desired humidity and CO_2_ sensors. Besides, these organic sensors exhibit relatively good characteristics without a heating process, leading to low power operation. To calibrate the temperature variation for the sensing polymers, in addition, a commercial thermistor is also exploited in the sensing system.

To fabricate the organic sensing materials, a p-type silicon-on-insulator (SOI) wafer is adopted as the device substrate for both developed humidity and CO_2_ sensors. After cleaning and drying procedures in a standard CMOS process by using nitrogen gas, the substrate is heated to completely remove humidity on the surface. The structure of the developed humidity and CO_2_ sensors includes two parallel electrodes and a sensing film in between. The physical dimensions of the parallel electrodes are defined by the photolithography with a gap (G) of 40 μm and a width (W) of 800 μm. The electrodes are deposited by using e-gun evaporation and the lift-off process with 20 nm/200 nm thick Cr/Au.

Sensing films for humidity and CO_2_ detection are intended to be spread in-between the parallel electrodes of the individual sensors by printing processes. The sensing film for humidity detection is composed of poly-3,4-ethylenedioxythiophene and poly-styrene -sulfonate (PEDOT:PSS) blended with aluminum–zinc oxide (AZO) [27]. The solvent for the sensing film is de-ionized (D.I.) water. Blending AZO nanoparticles increases the effective sensing area and improves material stability. The concentrations of PEDOT:PSS and AZO nanoparticles are 2.5 wt % and 0.0125 wt %, respectively. To improve material solubility and the degree of mixing, the solution is shaken with hot water in a sonicator for 1 h after all materials are blended. When the humidity sensor is exposed to moisture, the sensing film swells because of the absorption of water, resulting in the increment in conductive length [28]. Therefore, the humidity level can be sensed by measuring sensor resistance variation.

The room temperature operated polymer-based sensing material is the key to low power CO_2_ sensing. The characteristics of most polymer-based CO_2_ sensors depend on the reactions between the CO_2_ and amines in the backbone structure under the condition of high humidity. The existence of both humidity and CO_2_ result in the generation of bicarbonate doped in the sensing film, leading to higher conductivity. The sensing material for CO_2_ detection is prepared from the mixture of emeraldine base-polyaniline (EB-PANI) and PEDOT:PSS with a 1/1 ratio. The solutions of PEDOT:PSS and EB-PANI are first dissolved in D.I. water and in NMP (1-Methyl-2-pyrrolldinore) for 1 wt % solutions, respectively. Then, the PEDOT:PSS solution is added into the EB-PANI solution with stirring. The resultant solution is drop-casted on and in-between the fabricated micro-electrodes and is dried out in an oven at 60 °C with dynamic vacuum for 24 h to form the sensing film.

Since the developed CO_2_ sensor is specifically for indoor environmental monitoring, the sensing range is designed to be from 500 ppm to 10,000 ppm. According to the safety standard of indoor CO_2_ concentration from National Institute for Occupational Safety and Health (NIOSH), this sensing range covers different ventilation conditions, including the uncomfortable levels for human indoor activities. The designed organic sensor is cheaper than the commercial ones and needs much less power consumption. Therefore, it is more suitable for self-powered indoor environmental monitoring.

### 2.2. Design of the SoC Enabled Sensing Platform

**Figure 2:**
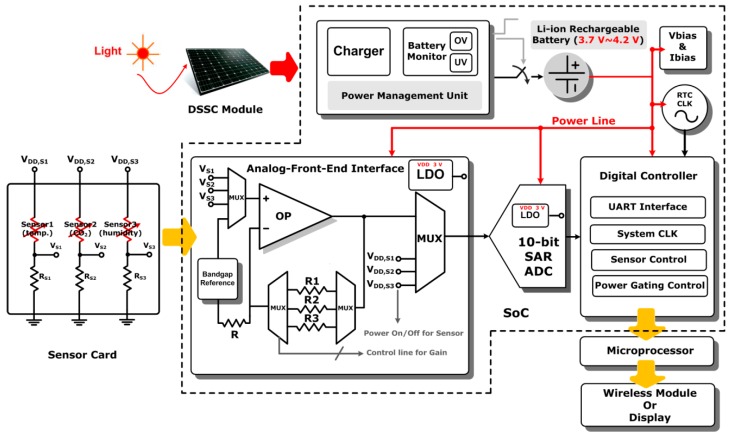
The block diagram of the designed system-on-chip (SoC) sensing platform with a replaceable sensor card.

The proposed SoC enabled sensing platform is designed to be powered by a DSSC module. The average power generation of the DSSC is around 1.2 mW under a typical indoor office lighting condition with 400 lux luminance level. The block diagram of the SoC as shown in Figure 2 is designed to collect environmental (temperature, CO_2_ and humidity) information under a stringent power budget. The SoC includes a power management unit (PMU), analog-front-end (AFE), an analog-to-digital converter (ADC), a digital control unit, a bandgap reference circuit and several low drop-out voltage regulators (LDO). An Universal Asynchronous Receiver/Transmitter (UART) interface with RS232 data format is employed in the SoC to export data to a microprocessor. Furthermore, a wireless module is employed so that the digitized sensing data can be transmitted to cloud servers and smart-phones for displaying.

The charger in the PMU transfers energies scavenged from the DSSC module to a Li-ion rechargeable battery. The schematic of the proposed PMU shown in Figure S2. The schematic of the charger circuit is shown in Figure S3 includes stabilize and charging sub-circuits. The charger starts with the constant current (CC) mode and switches to the constant voltage (CV) mode once the battery voltage achieves the desired level (around 3.7–4.2 V). When the battery voltage is higher than the desired level, e.g., more than 4.2 V, the charger should be disconnected from the battery to prevent the battery from being damaged. On the other hand, the charger should also be disconnected from the load if the battery voltage is lower than 2.7 V to prevent the collapse of the battery voltage. Therefore, it is necessary for the detection of the “Over-Voltage” (OV) and “Under-Voltage” (UV) to be implemented by the charger circuit. The schematic of the over voltage and under voltage detection circuit is shown in Figure S4. Finally, a power-on-reset (POR) circuit shown in Figure S5 is implemented in the PMU to reset digital registers during the system start-up phase. The charging process waveform of the Li-ion battery charger is shown in Figure S6. The measurement results of the battery detection circuit are depicted in Figure S7a,b, showing its function in monitoring the battery voltage. The waveforms of POR signal and supply voltage are shown in Figure S8. The employed operational amplifier (OPA) in the AFE adopts a folded-cascode topology with a p-channel MOSFET (PMOS) differential pair input stage biased in the subthreshold region and a class-AB output stage to drive resistive loads with high power efficiency. The ADC adopts successive approximation register (SAR) architecture for low power consumption with intended 10-bit resolution [24]. Through the UART interface, the digitized environmental data by the ADC can be sent to a low-power off-chip microprocessor and a commercially wireless module for further signal processing, displaying, or wireless communication.

**Figure 3:**
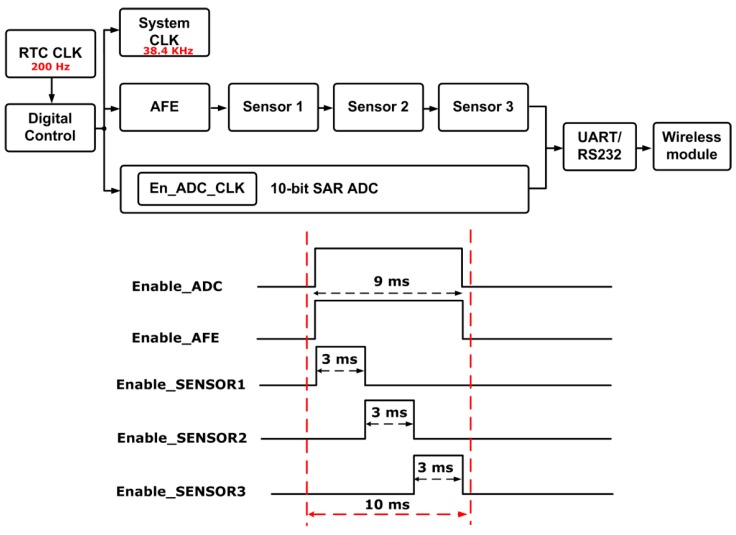
The control flow chart and the timing diagram.

Two clock sources are incorporated in the sensing state machine: a real-time clock (RTC, 200 Hz) for power gating control and a system clock (38.4 kHz) for internal state machine and for data transfer. The on-chip digital controller schedules the biasing and on-chip regulators to be turned on 1 ms earlier than the AFE and ADC circuits for settling. The AFE and ADC circuits are turned on afterward only for a short period of time (9 ms) to sense and to read out individual sensor information sequentially (3 ms for each sensor). Most of the time, these circuits are turned off with a very small duty cycle (10 ms/1 s = 0.01). The control flow chart and timing diagram are shown in Figure 3. In such a power-gating scheme, the average power consumption in operation is reduced significantly compared with the instantaneous power consumption when all the circuits are turned on, which can be calculated by the following equation:

(1) $$P_{\mathrm{avg}}{=P}_{\mathrm{instant}}\times D,$$

where P_avg_ is the average power consumption of the system, P_instant_ is the instantaneous power of the system, and D is the duty cycle in the power-gating scheme. The designed average power consumption of the sensing SoC is 3.2 μW, corresponding to 0.277 J for one day operation. In summary, a power gating control scheme with such a small duty cycle facilitates the designed SoC to achieve ultra-low power consumption.

### 2.3. Experimental Protocol

To characterize the performance of the developed environmental sensing platform, an LCR meter, Agilent 4980A, was used to characterize the sensing film impedance in different testing conditions. Before performing measurements, the ambient gas in the measuring chamber was first pumped out by a mechanical pump. Then, the dry air consisting of 20% oxygen and 80% nitrogen was injected into the chamber. The chamber was vacuumed again to the level of 10^−3^ torr as the initial condition. To set up the relative humidity test, the humidity formation was realized by water evaporation under different pressure settings. The relative humidity was continuously measured for 2 min. Between different tests of different humidity levels, the chamber was cleaned by following the previously described procedures to ensure the same initial condition. The humidity level was referenced to a commercial humidity meter with an embedded SHT11 humidity sensor chip, which was also placed in the chamber.

A wireless module and an electronic-ink display were connected to the sensing platform so that the measurement data could be obtained directly from the electronic-ink display or from a developed display webpage. The developed sensing platform was enclosed in a chamber in all the measurements to ensure that the air condition is under control.

## 3. Results and Discussion

### 3.1. Measured Characteristics of Humidity and CO_2_ Sensing Films

**Figure 4:**
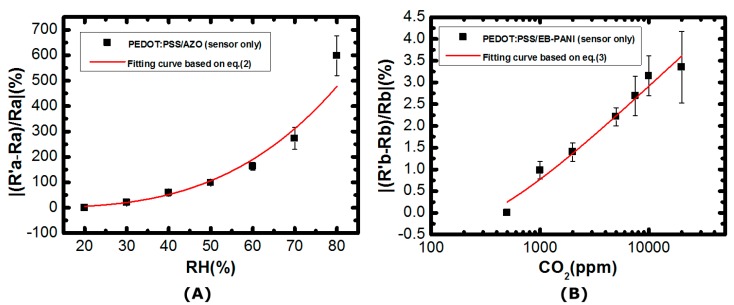
(**A**) The measured characteristics of the designed humidity sensor; (**B**) The measured characteristics of the designed CO_2_ sensor.

In the humidity measurements, the humidity level can be estimated by the following regression equation:

(2) $$\left|\frac{\left({R^{\prime }}_{a}-R_{a}\right)}{R_{a}}\right|\left(\%\right)={\mathrm{mRH}}^{n}$$

where RH represents the relative humidity level in percentage, *R_a_* is the resistance of the sensor that is exposed to the environment of 20% RH and ${R^{\prime }}_{a}$ is the measured resistances under different RH conditions. The extracted values of parameters of m and n are m = 3.95 × 10^−4^, and n = 3.196, respectively. The measured responses of the humidity sensor are shown in Figure 4A. The developed humidity sensor exhibits 20%–600% resistance change over the humidity level ranging from 30% RH to 80% RH. The comparisons between the designed humidity sensor and commercial products are tabulated in Table S1.

In the CO_2_ measurements, humidity is kept at 60% RH. The resistance responses of the sensor can be expressed by the following equation:

(3) $$\left|\frac{\left({R^{\prime }}_{b}-R_{b}\right)}{R_{b}}\right|\left(\%\right)=p-q*\ln\left(\left[{\mathrm{CO}}_{2}\right]+r\right)$$

where [CO_2_] is the CO_2_ concentration in ppm, *R_b_* is the resistance of the sensor when it is exposed to 500 ppm CO_2_, ${R^{\prime }}_{b}$ is the measured resistances at different CO_2_ concentrations. The extracted values of parameters p, q, and r are p = −6.488, q = −1.018, and r = 250, respectively. In Figure 4B, it is clear that the PEDOT:PSS/EB-PANI material-based sensor exhibits high sensitivity when the detection concentration ranges from 1000 ppm to 10,000 ppm. The corresponding sensor resistance varies from 0.98% to 3.15%. The comparisons between the designed CO_2_ sensor and commercial products are tabulated in Table S2.

### 3.2. Measurement Results from the Developed Sensing Platform

**Figure 5:**
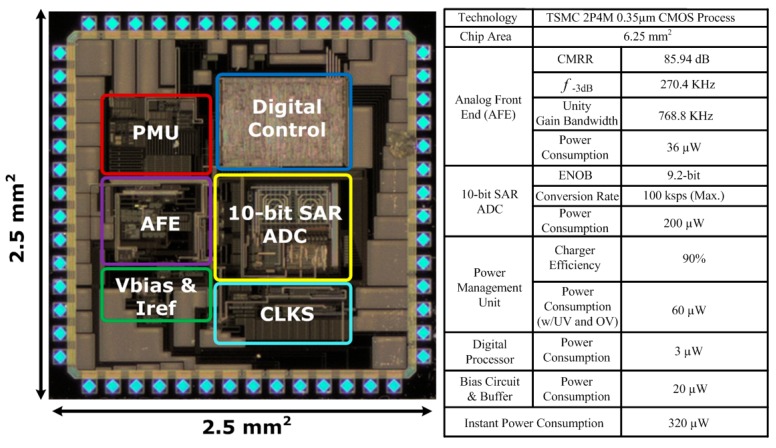
The die photo of the designed SoC and its measured performance.

**Figure 6:**
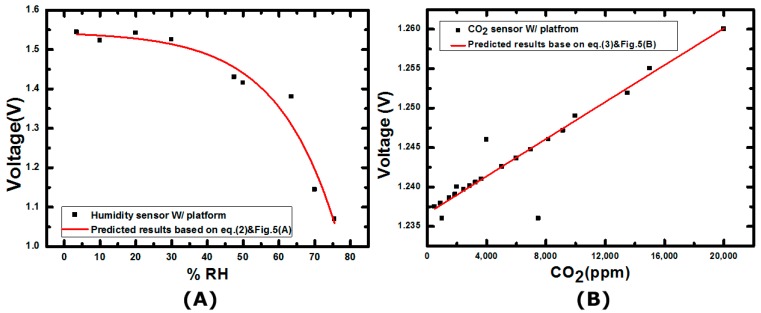
(**A**) Humidity and (**B**) CO_2_ concentration measurement results from the developed sensing platform.

**Figure 7:**
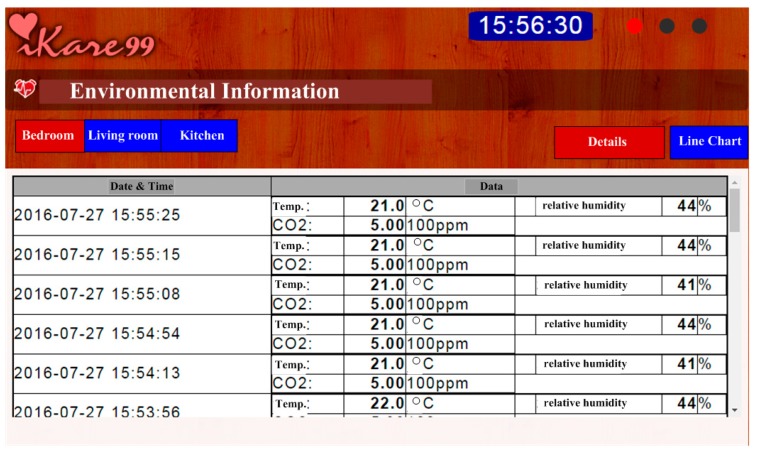
The measured data, including temperature, CO_2_ and humidity concentration, received from the developed indoor environmental sensing platform can be transferred to a cloud server and browsed from a website.

The SoC chip has been designed and fabricated in a standard 0.35 μm CMOS process. The die photo is shown in Figure 5, which occupies an area of 6.25 mm^2^ with testing pads. The performance summary of the designed SoC is also shown in Figure 5. The functionality of the developed environmental sensing platform is verified using similar experimental protocols that have been described earlier. The impedances of the developed organic sensors indicate humidity levels and CO_2_ concentrations. The impedance variations are converted into voltages by using a resistive voltage divider as shown in Figure 2. The sensed voltages are amplified by using a non-inverting amplifier configuration in the AFE block. The amplified signals are digitized by a 10-bit SAR ADC. The measured data is packed in RS232 format and then transmitted through the UART interface to a low-power off-chip MSP430 microprocessor, which is further connected to an electronic-ink display or a commercial wireless module. The humidity and CO_2_ concenrations are converted into voltages by the sensing platform as shown in Figure 6A,B, respectively, with the predicted results based on Equations (2) and (3) and Figure 4A,B, of which vertical axes have been re-scaled to voltages for convenience. The measured data of the three indoor air quality parameters, temperature, humidity, and CO_2_ has been transmitted to a cloud server and can be browsed on a smart phone or a computer as illustrated in Figure 7.

### 3.3. Self-Sustainability of the Proposed Sensing Platform

To verify the self-sustainability of the proposed sensing platform, a DSSC module with the total area of 240 cm^2^ was employed to measure the total energy that can be harvested in an indoor environment under the condition of illuminance level of 400 lux for 16 h in one day. The DSSC module was put in the dark for 8 h. The power harvested from the DSSC module is 1.2 mW during the period with indoor light and the average power is 880 μW in one day. Therefore, total energy of 76 J can to be harvested from the employed DSSC module in one day, which is sufficient for the developed multi-sensor platform (0.277 J). Compared with other previous works [29,30,31], the developed sensing system consumes the lowest power consumption and is self-sustained.

## 4. Conclusions

A self-sustained environmental indoor sensing platform is realized by a low-power SoC consuming average power of 3.2 μW and integrated with printable organic humidity and CO_2_ sensors. By adopting printable organic materials as the humidity and CO_2_ sensors, the fabrication costs can be significantly reduced. These sensors can operate at room temperature, achieving low power operation. The humidity sensor exhibits high sensitivity of 20%–600% resistance change corresponding to 30%RH–80%RH environmental humidity level variation. The PEDOT:PSS/EB-PANI-based CO_2_ sensor exhibits 0.98% to 3.15% resistance change corresponding to 1000 ppm to 10,000 ppm CO_2_ variation. Measurement results verify that the proposed sensing platform with multiple sensors can be self-sustained by scavenging indoor light energies via a DSSC module with an area of 240 cm^2^.

## Supplementary Materials

The following are available online at http://www.mdpi.com/1424-8220/17/4/715/s1, Figure S1. Temeratire measurement results from the developed sensing platform. Figure S2. Block diagram of the proposed power management unit. Figure S3. Schematic of the charger circuit. Figure S4. Schematic of the over voltage and under voltage detection circuit. Figure S5. (a) Schematic of the power-on-reset circuit; (b) timing diagram of the power-on-reset circuit. Figure S6. The charging process waveform of the charger. Figure S7. The battery monitoring circuit measurement results. Figure S8. The power-on-reset measurement results. Table S1. The comparison between the proposed humidity sensors and commercial products. Table S2. The comparison between the proposed CO_2_ sensors and commercial products.
