# Supplementary material for: A Self-Sustained Wireless Multi-Sensor Platform Integrated with Printable Organic Sensors for Indoor Environmental Monitoring
Source: Sensors (Basel). 2017 Mar 29;17(4):715. doi: 10.3390/s17040715 (PMC5421675; doi:10.3390/s17040715)
Supplement: Supplementary file 1 [file sensors-17-00715-s001.pdf]

Supplementary Materials: A Self-Sustained Wireless Multi-Sensor Platform Integrated with Printable Organic Sensors for Indoor Environmental Monitoring

Chun-Chang Wu, Wen-Yu Chuang, Ching-Da Wu, Yu-Cheng Su, Yung-Yang Huang, Yang-Jing Huang, Sheng-Yu Peng, Shih-An Yu, Chih-Ting Lin and Shey-Shi Lu

1. Temperature Sensor Measurement Results

The waveform of temperature is followed by a linear equation from 20 °C to 70 °C. The measurement results are shown in Figure S1.

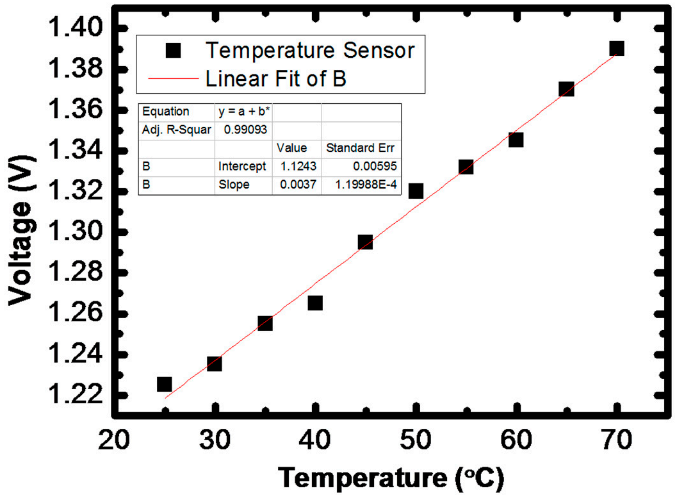

Figure S1. Temeratire measurement results from the developed sensing platform.

The comparisons between the designed humidity sensor and commercial products are tabulated in Table S1.

Table S1. The comparison between the proposed humidity sensors and commercial products.

Model No.	HR31	DHT11	H25K5	This Work
Detection range (RH)	20%–95%	20%–90%	<90%	20%–80%
Sensing (principle)	Polymer based humidity-sensitive resistor	Resistive humidity sensor	Resistive humidity sensor	Polymer based PEDOT:PSS
Power consumption (mW)	0.2<br>1.5 VAC(Max. sine)	0.6<br>3.25-5.5 VDC	0.26<br>1 VrmsAC	0.01<br>1 VDC
Size (mm^3)	7.3 × 11.3 × 3.5	12 × 15.5 × 5.5	6.8 × 12.8 × 3.3	15 × 15 × 15
Cost (USD)	2.5	2.2	3.5	1.5

The comparisons between the designed CO_2 sensor and commercial products are tabulated in Table S2, which indicates a significant improvement attained by the proposed polymer sensors for low power operation.

Table S2. The comparison between the proposed CO_2 sensors and commercial products.

Model No.	Figaro (CDM4161A)	CO2Meter (COZIR)	MG811 (CO_2)	This Work
Detection range (ppm)	400–4000	2000–10,000 or 0–2000	350–10,000	500–20,000
Sensing (principle)	TGS4161 (solid state eletrolyte)	GSS IR LED absorption	Solid electrolyte cell	Polymer based (PEDOT:PSS/ EB-PANI)
Power consumption (mW)	300	3.5	1200	~0.005 (1V DC)
Size (mm^3)	45 × 26 × 17	40 × 40 × 20	19 × 19 × 23	15 × 15 × 15
Cost (USD)	79	109	35	~3

2. Power Management Unit

The schematic of the proposed power management unit is shown in Figure S2. A battery charger is implemented to charge a Li-ion battery with the power harvested from a commercial dye-sensitized solar cell (DSSC) module with indoor light at 400lux. The schematic of the charger circuit is shown in Figure S3 includes stabilize and charging sub-circuits. The stabilize sub-circuit is composed of a current mirror (M_1 and M_2),a pass transistor (M_3), an error amplifier (OTA) used in the constant current (CC) mode, and a current source that is formed by a series of diode connected transistors (M_4, M_5, and M_6).The charging sub-circuit includes another current mirror (M_8 and M_{10}), a transistor switch (M_9), one error amplifiers (OP1) in the constant voltage (CV) mode and one amplifier (OP2) as a comparator that is used to switch the charger between the CC and the CV modes. The charging circuit starts with the CC mode and switches to the CV mode once the battery voltage, V_{BAT}, is high enough (around 3.7–4.2 V).

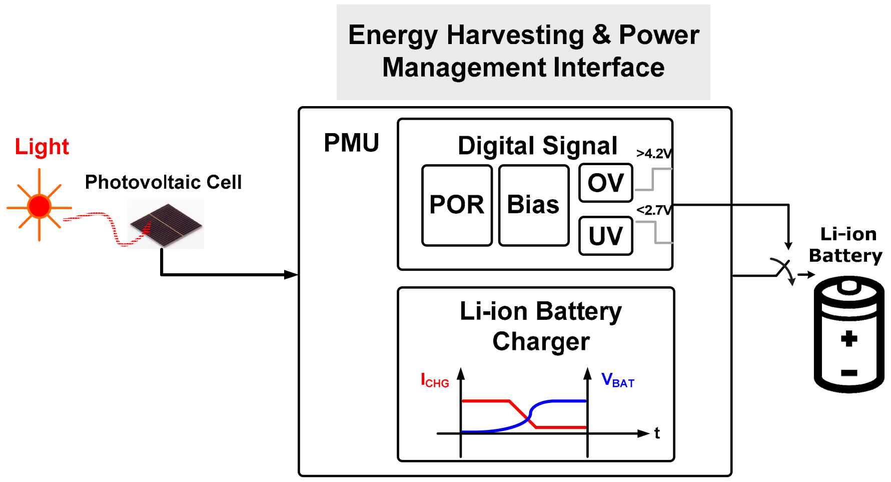

Figure S2. Block diagram of the proposed power management unit.

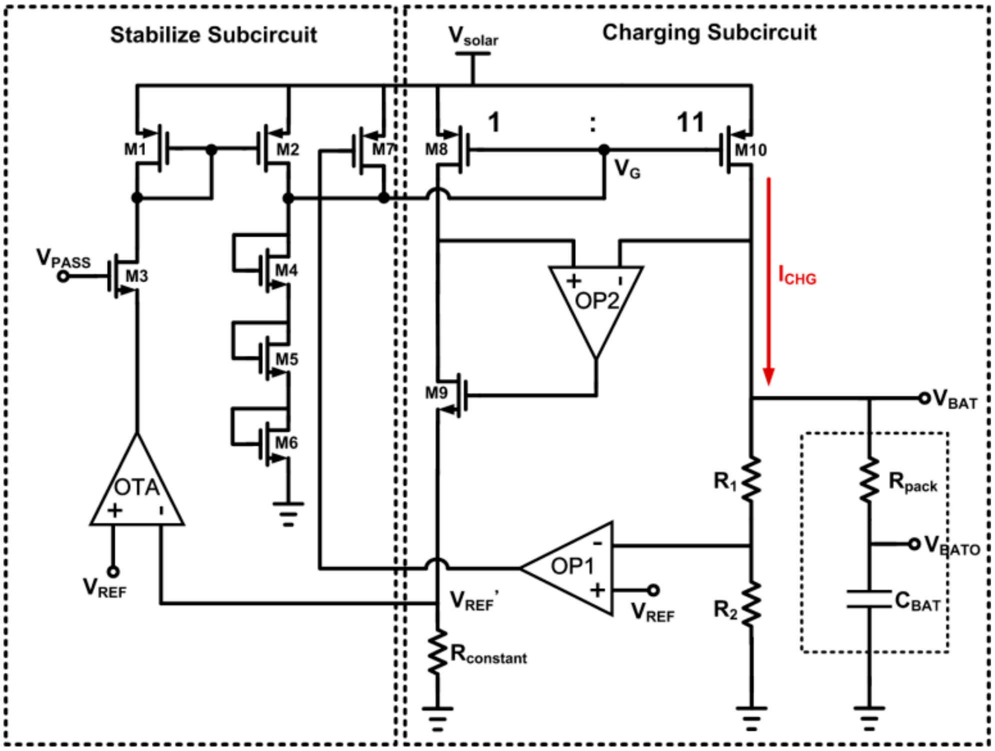

Figure S3. Schematic of the charger circuit.

When the battery voltage is too high (more than 4.2 V), the charger should be disconnected from the battery to prevent the battery from being damaged. On the other hand, the charger should also be disconnected from the load if the battery voltage is too low (less than 2.7 V) to prevent the collapse of the battery voltage. Therefore, the detection of the "Over-Voltage" (OV) and "Under-Voltage"(UV) is necessary, which is implemented by the circuit shown in Figure S4.

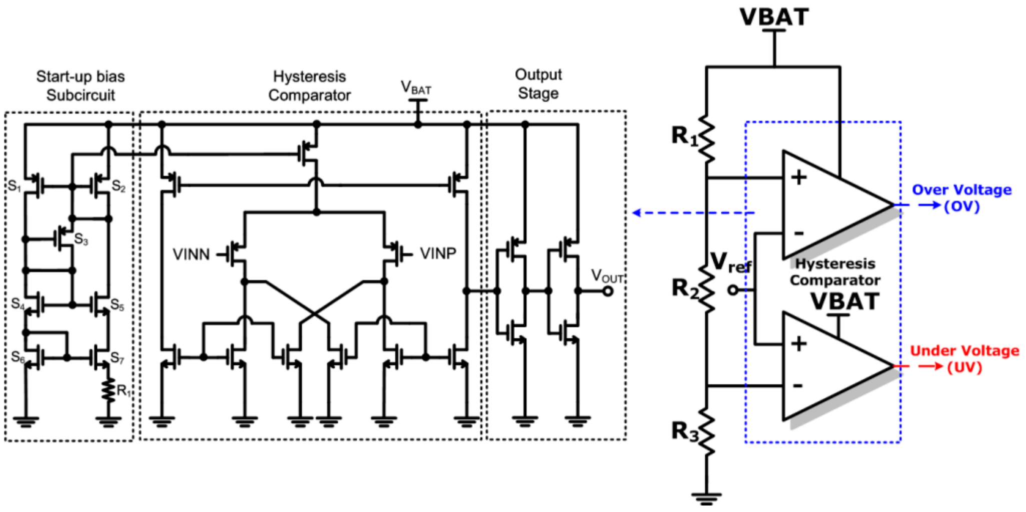

Figure S4. Schematic of the over voltage and under voltage detection circuit.

Finally, a power-on-reset (POR) circuit shown in Figure S5 is implemented in the Power Management Unit (PMU) to reset digital registers during the system start-up phase. The POR circuit is composed of RC-charging, timing delay, and inverter chain sub-blocks. After the supply voltage is activated, the POR output voltage changes to high logic level with some delay depending on the charging speed of the Metal-Oxide-Semiconductor (MOS) capacitors.

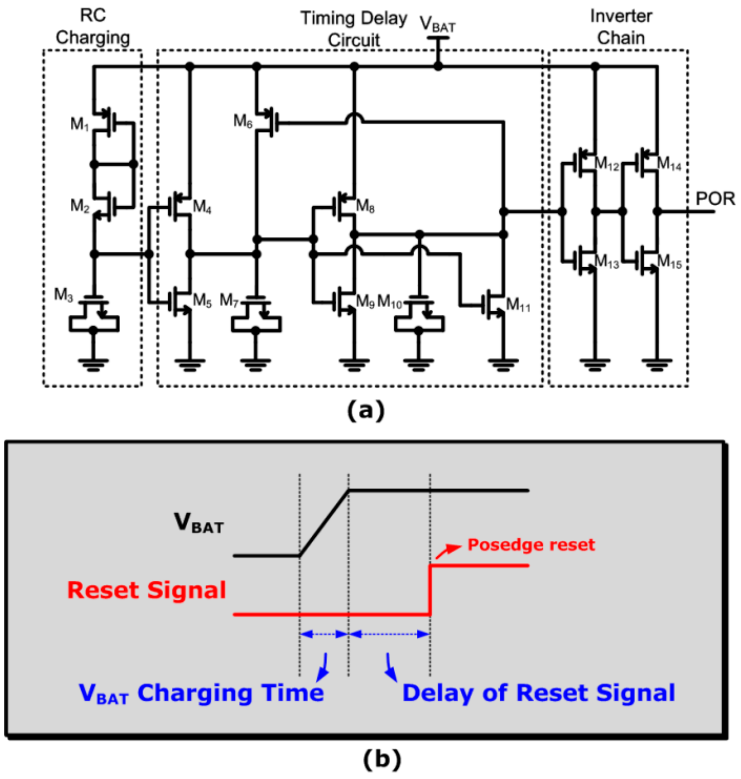

Figure S5. (**a**) Schematic of the power-on-reset circuit; (**b**) timing diagram of the power-on-reset circuit.

The charging process waveform of the Li-ion battery charger is shown in Figure S6. It is easily to distinguish the change of constant current mode (CC) and constant voltage mode (CV) through observing the waveform of the charging process.

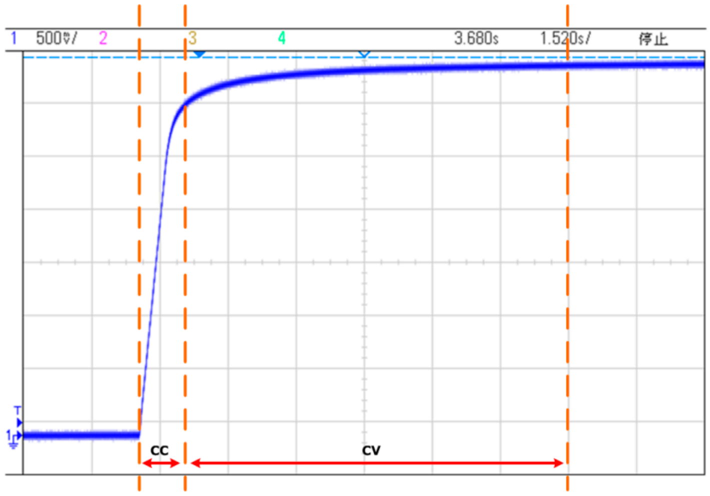

Figure S6. The charging process waveform of the charger.

The measurement results of the battery detection circuit are depicted in Figure S7a,b, showing its function in monitoring the battery voltage. When the voltage of the battery over 4.2 V, an "OV" positive edge warning signal is sent to digital controller. On the other hand, the digital controller receives an "UV" positive edge warning signal, when the voltage of the battery under 2.7 V. The waveforms of POR signal (number 2) and supply voltage (number 3) are shown in Figure S8. After the supply voltage is activated, the POR output voltage changes to high logic level for about 20 μs delay time.

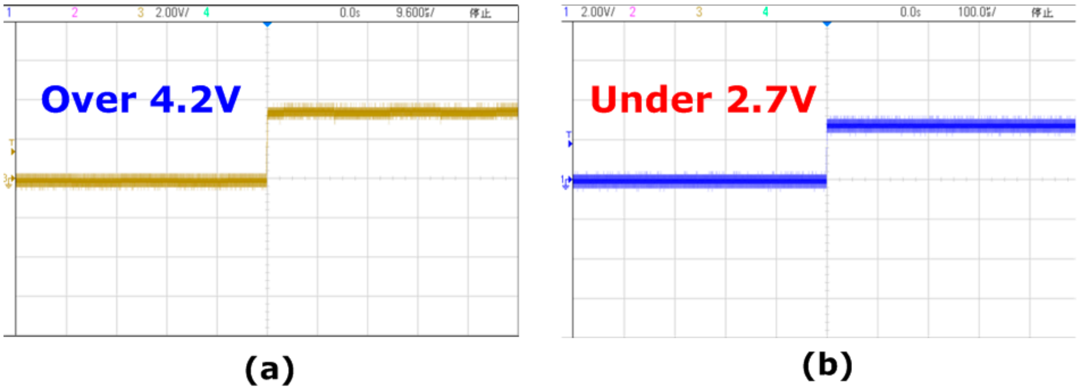

Figure S7. The battery monitoring circuit measurement results.

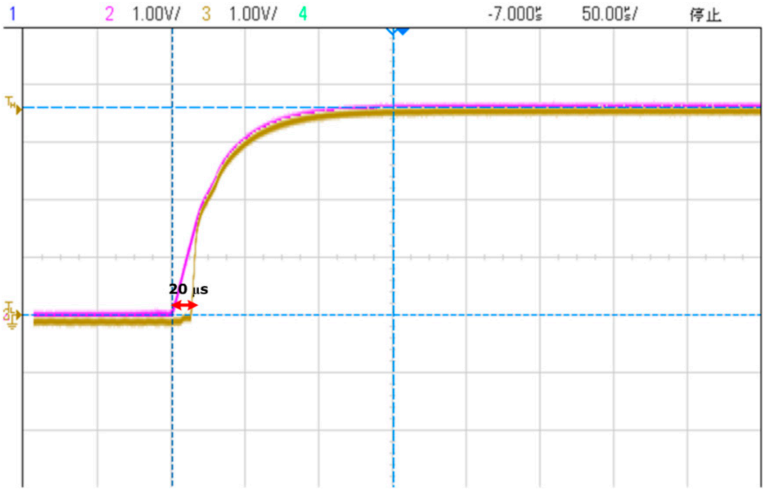

Figure S8. The power-on-reset measurement results.
